# BspR/BtrA, an Anti-σ Factor, Regulates the Ability of *Bordetella bronchiseptica* To Cause Cough in Rats

**DOI:** 10.1128/mSphere.00093-19

**Published:** 2019-04-24

**Authors:** Keiji Nakamura, Noriko Shinoda, Yukihiro Hiramatsu, Shinya Ohnishi, Shigeki Kamitani, Yoshitoshi Ogura, Tetsuya Hayashi, Yasuhiko Horiguchi

**Affiliations:** a Department of Molecular Bacteriology, Research Institute for Microbial Diseases, Osaka University, Suita, Osaka, Japan; b Department of Bacteriology, Faculty of Medical Sciences, Kyushu University, Fukuoka, Japan; University of Kentucky

**Keywords:** *Bordetella*, BspR/BtrA, cough

## Abstract

Bordetella pertussis, B. parapertussis, and B. bronchiseptica cause respiratory infections, many of which are characterized by coughing of the infected hosts. The pathogenesis of the coughing remains to be analyzed, mainly because there were no convenient infection models of small animals that replicate coughing after *Bordetella* infection. Here, we present a coughing model of rats infected with B. bronchiseptica. Rats, which are one of natural hosts of B. bronchiseptica, were readily infected with the organisms and showed frequent coughing. B. pertussis also caused coughing in rats, which is consistent with previous reports, but the cough response was less apparent than the B. bronchiseptica-induced cough. By using the rat model, we demonstrated that adenylate cyclase toxin, dermonecrotic toxin, and the type III secretion system are not involved in cough production, but BspR/BtrA (different names for the same protein), an anti-σ factor, regulates the production of unknown factor(s) to cause coughing. Rat coughing was observed by inoculation of not only the living bacteria but also the bacterial lysates. Infection with *bspR* (*btrA*)-deficient strains caused significantly less frequent coughing than the wild type; however, intranasal inoculation of the lysates from a *bspR* (*btrA*)-deficient strain caused coughing similarly to the wild type, suggesting that BspR/BtrA regulates the production of the cough factor(s) only when the bacteria colonize host bodies. Moreover, the cough factor(s) was found to be heat labile and produced by B. bronchiseptica in the Bvg^+^ phase. We consider that our rat model provides insight into the pathogenesis of cough induced by the *Bordetella* infection.

**IMPORTANCE** Whooping cough is a contagious respiratory disease caused by Bordetella pertussis. This disease is characterized by severe paroxysmal coughing, which becomes a heavy burden for patients and occasionally results in death; however, its pathogenesis remains largely unknown. The major obstacle to analyzing *Bordetella*-induced coughing is the lack of conventional animal models that replicate coughing. As Bordetella pertussis is highly adapted to humans, infection models in experimental animals are not considered to be well established. In the present study, we examined coughing in rats infected with B. bronchiseptica, which shares many virulence factors with B. pertussis. Using this rat model, we demonstrated that some of the major virulence factors of *Bordetella* are not involved in cough production, but an anti-σ factor, BspR/BtrA, of B. bronchiseptica regulates the production of unknown cough-causing bacterial factor(s). Our results provide important clues to understand the mechanism by which *Bordetella* induces cough.

## Abstract (precis)

Whooping cough is a contagious respiratory disease caused by Bordetella pertussis. This disease is characterized by severe paroxysmal coughing, which becomes a heavy burden for patients and occasionally results in death; however, its pathogenesis remains largely unknown. The major obstacle to analyzing *Bordetella*-induced coughing is the lack of conventional animal models that replicate coughing. As Bordetella pertussis is highly adapted to humans, infection models in experimental animals are not considered to be well established. In the present study, we examined coughing in rats infected with B. bronchiseptica, which shares many virulence factors with B. pertussis. Using this rat model, we demonstrated that some of the major virulence factors of *Bordetella* are not involved in cough production, but an anti-σ factor, BspR/BtrA, of B. bronchiseptica regulates the production of unknown cough-causing bacterial factor(s). Our results provide important clues to understand the mechanism by which *Bordetella* induces cough.

## INTRODUCTION

Whooping cough is a highly contagious respiratory disease that is caused by the pathogenic bacterium Bordetella pertussis. The disease is characterized by a wide range of clinical manifestations, including leukocytosis, hypoglycemia, bronchopneumonia, and severe paroxysmal coughing. The disease is preventable by vaccination; however, the number of cases is increasing, probably because of the infection of adolescents and the prevalence of antigen-mutated strains (https://www.who.int/immunization/monitoring_surveillance/burden/vpd/surveillance_type/passive/pertussis/en/, https://www.cdc.gov/pertussis/surv-reporting.html) (1–4). The presentation of whooping cough usually proceeds in catarrhal, paroxysmal, and convalescent phases. Notably, coughing in the paroxysmal phase becomes a heavy burden for patients, but its pathogenesis is totally unknown, and effective therapeutic methods have not been developed: macrolide antibiotics, which are used to eradicate the bacteria, do not ease the coughing of patients in the paroxysmal phase. General antitussive drugs are also ineffective. Therefore, a therapeutic method specific to B. pertussis-caused coughing needs to be developed on the basis of understanding its pathogenesis.

The major obstacle to the study of *Bordetella*-caused coughing is the lack of convenient animal models that replicate coughing after *Bordetella* infection in humans (5). In 1939, Hornibrook and Ashburn described rats that developed coughing or sneezing paroxysms after intranasal inoculation of B. pertussis (6). These results were reconfirmed 50 years later by two distinct groups (7–13). However, these researchers did not analyze the mechanism of coughing, and their rat models have not been utilized by other researchers, probably because of the complex procedures in which the bacteria were embedded in agarose beads and introduced into a bronchus that was surgically exposed. Although infection of rats with living B. pertussis without the beads was also attempted, rats barely exhibited coughing (7). Mice have been widely used to examine numerous aspects of *Bordetella* infection; however, it has been pointed out that mice do not cough (5). Piglets and baboons were recently reported as animal models for whooping cough (14, 15). These animals replicated many symptoms of whooping cough, including paroxysmal cough, but it may be difficult to use sufficient numbers of these large animals (or primates) to analyze cough mechanisms because of ethical and cost issues. Infection models of small and convenient laboratory animals exhibiting cough have long been awaited.

Since B. pertussis is highly adapted to humans, experimental infection models of this organism are not considered to be well established. B. parapertussis and B. bronchiseptica, in addition to B. pertussis, are known as the classical *Bordetella* spp. causing respiratory infections in humans and other mammals (16–18). *B. parapertussis*, which is known to cause a whooping cough-like disease in humans, has two distinct lineages that infect humans and sheep, respectively. B. bronchiseptica causes chronic respiratory diseases in a wide range of mammals, including pigs, dogs, and various laboratory animals. Coughing is commonly observed in natural infections of classical *Bordetella* with a few exceptions (18). The members of classical *Bordetella* share many virulence factors, the expression of which is largely regulated by the BvgAS two-component system (19). When *Bordetella* is grown at 37°C or in the absence of MgSO_4_ and nicotinic acid, the BvgAS system activates transcription of a set of *vag* (*vir*-activated gene), including various virulence genes. When the organisms are grown at a temperature of less than 26°C or in the presence of MgSO_4_ or nicotinic acid at 37°C, the BvgAS system becomes inactive and the transcription of *vag* is shut down. The former bacterial state is called the Bvg^+^ phase, and the latter state is called the Bvg^–^ phase. This characteristic phase conversion is common in classical *Bordetella*. Therefore, analyses of cough caused by classical *Bordetella* other than B. pertussis may provide clues to understanding the pathogenesis of B. pertussis-induced cough. In this study, we chose B. bronchiseptica as a test organism, looked for a satisfactory animal model replicating the *Bordetella*-induced coughing, and rediscovered that rats coughed after intranasal inoculation of B. bronchiseptica. Using this model, we demonstrate that some of the major virulence factors of *Bordetella*, such as adenylate cyclase toxin, dermonecrotic toxin, and type III secretion effectors, do not contribute to cough production but that a recently identified anti-σ factor, BspR/BtrA (different names for the same protein) of B. bronchiseptica is a key regulator to produce (an) unknown cough-causing bacterial factor(s).

## RESULTS

### *Bordetella* infection in rats.

**FIG 1:**
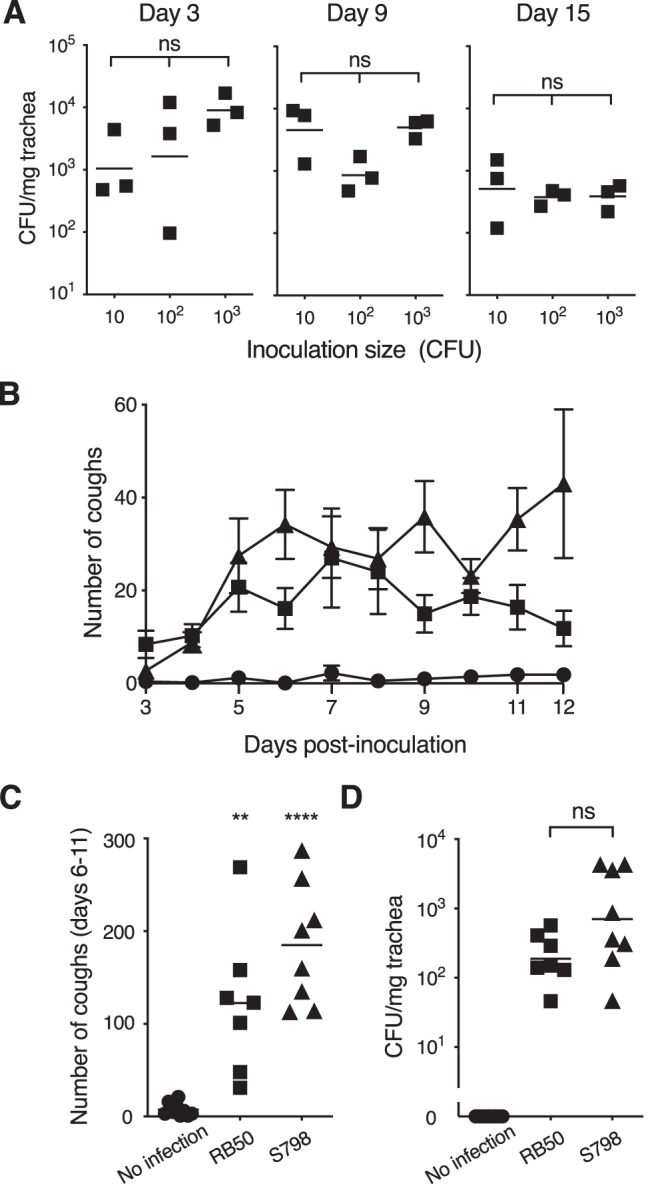
Coughing of rats infected with B. bronchiseptica. (A) Colonization of B. bronchiseptica in the rat trachea. The indicated number of B. bronchiseptica RB50 was intranasally inoculated into rats, and the bacteria recovered from tracheas were counted on days 3, 9, and 15. The number of CFU per mg of trachea is shown for each rat. Bars represent geometric means. (B and C) Number of coughs from rats infected with B. bronchiseptica. Rats were intranasally inoculated with 10^3^ CFU of B. bronchiseptica RB 50 (filled squares, *n* = 7), S798 (filled triangles, *n* = 8), or SS medium without the bacteria (filled circles, *n* = 9). The numbers of coughs were counted for 5 min/rat/day on the indicated days (B) and added for 6 days from days 6 to 11 (C). (D) The numbers of bacteria recovered from tracheas were measured on day 15 postinoculation. Each plot in panel B represents the mean ± the standard error. Each horizontal bar in panels C and D represents the mean and the geometric mean, respectively. Symbols for significant levels indicate comparison with the control “No infection” (C) or between test groups indicated by brackets (D). ns, no significant differences.

In order to find a satisfactory animal model that replicates coughing or cough-like symptoms in response to intranasal infection by B. bronchiseptica, we reexamined a variety of small laboratory animals such as guinea pigs, mice, and rats. Guinea pigs were readily infected with the bacterium but did not exhibit coughing. Mice were found to be least sensitive to B. bronchiseptica infection among the tested animals and did not exhibit coughing either. In contrast, rats were readily colonized by a low dose of B. bronchiseptica (Fig. 1A), as reported previously (20, 21). The amount of the bacteria recovered from rat tracheas were not dependent on the inoculation size, indicating that the lower dose of bacterium was sufficient to establish infection in rats. In addition, rats responded to B. bronchiseptica infection with coughing postures (see Movie S1 in the supplemental material). The similar observation was previously reported as “sneezing” but was not analyzed in detail (21). We considered this cough-like posture to require further analyses and tentatively designated it as “cough.” When two distinct B. bronchiseptica isolates from a rabbit (RB50) and a pig (S798) were applied to the rat model, coughing was seen as early as 2 to 3 days after bacterial inoculation and persisted at least for 12 days (Fig. 1B). The total cough numbers from days 6 to 11 when coughing was most frequently exhibited were significantly higher than those of the noninfected group (Fig. 1C). The bacteria were recovered from rat tracheas at 15 days postinfection, demonstrating stable colonization of the inoculated bacteria (Fig. 1D).

10.1128/mSphere.00093-19.3 MOVIE S1 Rat’s coughing 7 days after inoculation of 10^3^ CFU of B. bronchiseptica. Download Movie S1, MOV file, 11.0 MB. Copyright © 2019 Nakamura et al. 2019 Nakamura et al. This content is distributed under the terms of the Creative Commons Attribution 4.0 International license.

### Rat coughing was not attributable to major virulence factors.

**FIG 2:**
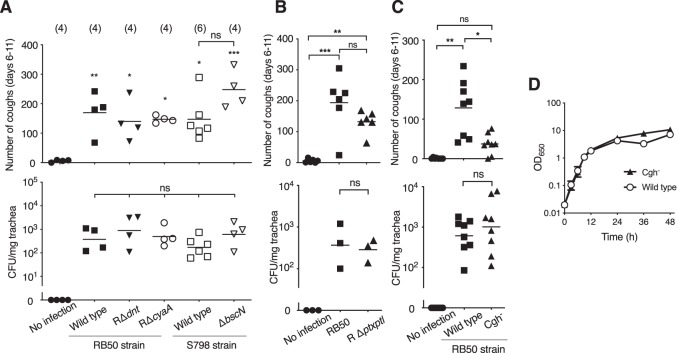
Coughing by rats infected with mutant strains of B. bronchiseptica. (A, B, and C, upper panels) Cough production by B. bronchiseptica and its derivatives. Rats were intranasally inoculated with 10^3^ CFU (A and C) or 10^2^ CFU (B) of the bacteria. Coughs by rats were counted for 5 min/rat/day for 6 days from days 6 to 11 postinoculation, and the total numbers of coughs are shown. (A, B, and C, lower panels) The numbers of bacteria recovered from rat tracheas on day 15 are shown. (A) The number of rats in each group is shown in parentheses. (B) Six mice for each group were inoculated, and three of them were sacrificed for enumeration of the bacteria on day 15. (C) Eight mice for each group were examined. (D) Growth curves of B. bronchiseptica wild-type and Cgh^–^ strains. B. bronchiseptica RB50 and its spontaneous mutant Cgh^–^ precultured in SS medium (*n* = 3) were diluted in the same medium to give an OD_650_ value of 0.02, followed by incubation at 37°C with shaking. Bacterial growth was evaluated by determining the OD_650_ values measured at the indicated periods of incubation. Each horizontal bar in the upper panels of A to C and in the lower panels represents the mean and the geometric mean, respectively. Symbols for significant levels indicate comparison with the control “No infection” (A) or between test groups indicated by brackets. ns, no significant differences.

It has been often pointed out that a virulence factor(s) involved in cough production should be shared by classical *Bordetella*, B. pertussis, B. parapertussis, and B. bronchiseptica, because they all cause coughing as a symptom in infected animals in various levels (18). Major virulence factors common in classical *Bordetella* are represented by adenylate cyclase toxin, dermonecrotic toxin, and type III-secreted effectors. We examined whether these virulence factors are involved in cough production using B. bronchiseptica mutant strains deficient in each factor. All B. bronchiseptica mutants derived from either RB50 or S798 colonized rat tracheas similarly to their parental wild-type strains and caused cough in infected rats (Fig. 2A), indicating that these common virulence factors are not attributable to cough production. Rats infected with S798 (*ΔbscN*), which is deficient in type III secretion, tended to exhibit more frequent coughing during the period of counting; however, the difference was statistically nonsignificant. Pertussis toxin, which is specifically produced by B. pertussis, is often discussed as the most probable cough-causing factor. B. bronchiseptica also carries the pertussis toxin genes *ptx-ptl* that are known to be silent. We examined the possibility that pertussis toxin, if produced by B. bronchiseptica, is involved in the cough production by applying a B. bronchiseptica *ptx-ptl* deletion mutant, RΔ*ptxptl*, to the rat coughing model. As shown in Fig. 2B, RΔ*ptxptl* also caused coughing, demonstrating that pertussis toxin does not play a role in cough production even if produced by B. bronchiseptica.

Thus, the cough factor was not found among the major known virulence factors. However, in the course of these experiments requiring different mutant strains, we unexpectedly isolated a spontaneous B. bronchiseptica RB50 mutant that proliferated similarly to the wild-type strain *in vitro* but exhibited reduced ability to induce coughing (Fig. 2C and D). This clone designated Cgh^–^ (**C**ou**gh^–^**) colonized rat tracheas equivalently to the wild-type strain, implying that Cgh^–^ has a mutation(s) in some gene(s) responsible for cough production. In addition, these results indicate that the rat coughing observed in the present study does not merely result from nonspecific host responses to respiratory bacterial infections.

### Involvement of BspR/BtrA in cough production.

**FIG 3:**
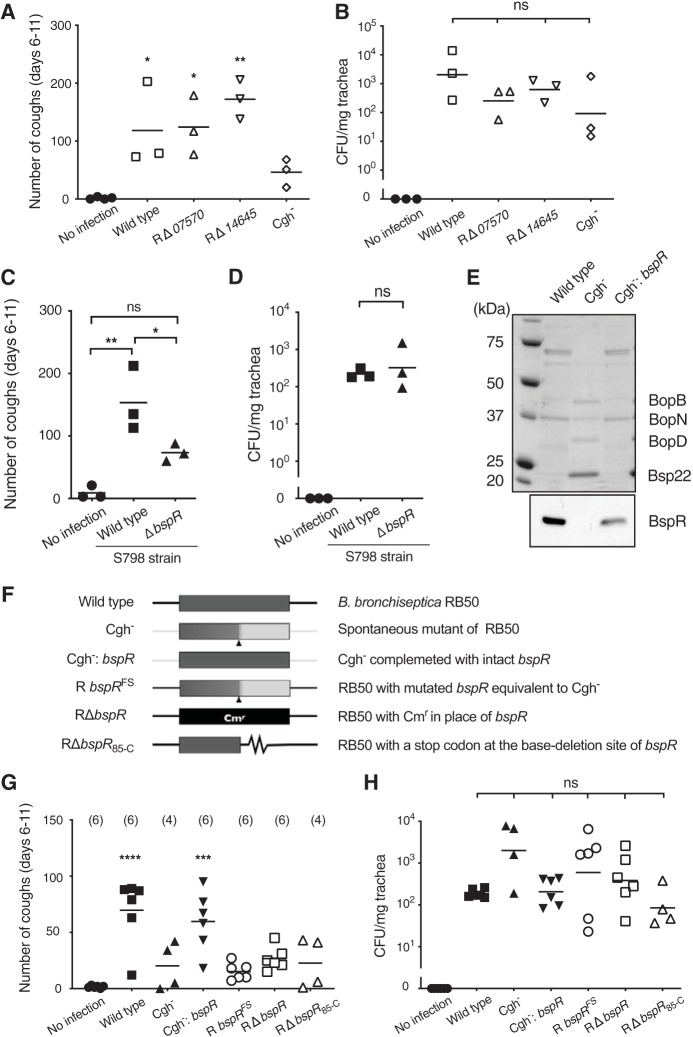
Involvement of *bspR* (*btrA*) in cough production in rats infected with B. bronchiseptica. Rats were intranasally inoculated with 10^3^ CFU of B. bronchiseptica RB50, S798, or their derivatives in 50 µl of SS medium. (A, C, and G) Coughs by rats were counted for 5 min/rat/day for 6 days from days 6 to 11 postinoculation, and the total numbers of coughs are shown. (B, D, and H) Numbers of bacteria recovered from rat tracheas on day 15. (E) Expression of effectors of the type III secretion system in Cgh^–^. SDS-PAGE analysis of culture supernatants of B. bronchiseptica mutants was performed (upper panel), as well as immunoblotting of whole-cell lysates for BspR (lower panel). (F) Schematic representation of *bspR* (*btrA*) with different mutations. Cgh^–^ has a spontaneous single base deletion (arrowhead) in *bspR*. Cgh^–^:*bspR* has an intact *bspR* gene with the chromosomal background of Cgh^–^. R*bspR*^FS^ has *bspR* with the single base deletion (arrowhead) with a wild-type chromosomal background. RΔ*bspR* has a chloramphenicol resistance gene substituted for intact *bspR*. RΔ*bspR*_85-C_ has a truncated *bspR* gene in which the stop codon was inserted at codon position 85. In complementation experiments, intact genes were provided by genomic integration to the corresponding position because plasmids for complementation were not maintained in bacterial cells for prolonged infection periods. Three rats from each test group were used for panels A to D. For panels G and H, the numbers of rats in each test group are shown in parentheses. Each horizontal bar represents the mean (A, C, and G) or the geometric mean (B, D, and H). Symbols for significant levels indicate comparison with the control “No infection” (A and G) or between test groups indicated by brackets (B, C, D, and H). ns, no significant differences.

To identify the mutated gene responsible for the reduced ability of Cgh^–^ to cause cough, we sequenced the complete genome of Cgh^–^ and searched for mutations specifically occurring in Cgh^–^ by comparison with the sequence of parental strain RB50. As a result, we found the following three distinct mutations: a single base (G) insertion in *BB_RS07570* (old locus tag = BB1518); a single base (C) deletion in *BB_RS08175* (old locus tag = BB1639), which had been reported as *bspR* or *btrA* (22, 23); and a nonsynonymous base substitution (C to A) in *BB_RS14645* (old locus tag = BB2918). Mutant strains with deletion in *BB_RS07570* (RΔ*07570*) or *BB_RS14645* (RΔ*14645*) were generated from RB50 and examined for cough production in the rat model (Fig. 3A and B). Both mutants colonized rat tracheas and produced cough to an extent comparable to the wild type. In contrast, a *bspR* (*btrA*)-deficient mutant of strain S798 (Δ*bspR*; a gift from A. Abe) caused significantly less frequent coughing than did the S798 wild type (Fig. 3C) but colonized similarly (Fig. 3D). The same results were obtained with a *bspR* (*btrA*)-deficient strain derived from strain RB50 (RΔ*bspR*; Fig. 3F, G, and H). Previously, *bspR* (*btrA*) was reported to be an anti-σ factor that negatively regulates the expression of genes on the loci of type III secretion system (22), and the deletion of *bspR* resulted in excessive secretion of type III effectors (23, 24). Cgh^–^ secreted a larger amount of effectors than the parental strain as revealed by SDS-PAGE (Fig. 3E), indicating that Cgh^–^ and the *bspR*-deficient strain share a similar phenotype in terms of the secretion of the type III effectors. We experimentally clarified the start codon of *bspR* (*btrA*), which had been inconsistently predicted (22–24) and found that BspR/BtrA consists of 191 amino acids (see Fig. S1 in the supplemental material), which was in agreement with the prediction by Kurushima et al. (23). Consequently, Cgh^–^ was found to have a C (cytosine) deletion at the base position of 253 in the open reading frame for *bspR* (*btrA*), which results in a frameshift at Leu^85^ (Fig. S1). Derivatives of RB50 with various mutations in *bspR* (*btrA*) were generated, as shown in Fig. 3F, and examined for tracheal colonization and cough production. R*bspR*^FS^, RΔ*bspR*, and RΔ*bspR*_85-C_, all of which have different types of mutations in *bspR* (*btrA*), colonized the trachea to an extent similar to the wild-type RB50 but barely caused coughing (Fig. 3G and H). In contrast, the *bspR* (*btrA*)-complemented Cgh^–^ (Cgh^–^:*bspR*), which produced a reduced amount of the type III effectors (Fig. 3E), induced coughing to a degree comparable to the wild type (Fig. 3G and H). Thus, we concluded that BspR/BtrA is, directly or indirectly, involved in causing coughing.

10.1128/mSphere.00093-19.4 FIG S1 Determination of the start codon of *bspR* (*btrA*). (A) Base and deduced amino acid sequences of *bspR* (*btrA*). Two ATGs are present 105 bp apart from each other in the 5′ region of *bspR* (*btrA*) open reading frame (underlined). The stop codons are indicated by asterisks. Cgh^–^ has a cytosine deletion at the position indicated by the arrow, resulting in a translational frameshift at Leu^85^, as shown in blue letters. (B) The B. bronchiseptica RB50 Δ*bspR* strain was transformed with pMIN136TDE-P*cyaA-*P*_bspR_-bspR*, pMIN136TDE-P*cyaA-*P*_bspR_-bspR*_ATG1_ (BspR_ATG1_), or pMIN136TDE-P*cyaA-*P*_bspR_-bspR*_ATG2_ (BspR_ATG2_) and cultivated for 5 h in SS medium. Whole-cell lysates were obtained and subjected to SDS-PAGE, followed by immunoblotting for BspR/BtrA, as described in Materials and Methods. The above-mentioned plasmids express BspR under the control of the promoters of *cyaA* and *bspR* (*btrA*). The former plasmid encodes intact *bspR*. The latter two plasmids encode *bspR* (*btrA*) with ACG in place of the second ATG and the first ATG, respectively. pMIN136TDE-P*cyaA* was used as a vector control. Note that BspR_ATG2_, but not BspR_ATG1_, produced BspR on immunoblotting, demonstrating that the second ATG functions as the start codon for BspR/BtrA. FtsZ was detected by anti-FtsZ as an internal control. Download FIG S1, PDF file, 0.2 MB. Copyright © 2019 Nakamura et al. 2019 Nakamura et al. This content is distributed under the terms of the Creative Commons Attribution 4.0 International license.

### Cough production by bacterial lysates.

**FIG 4:**
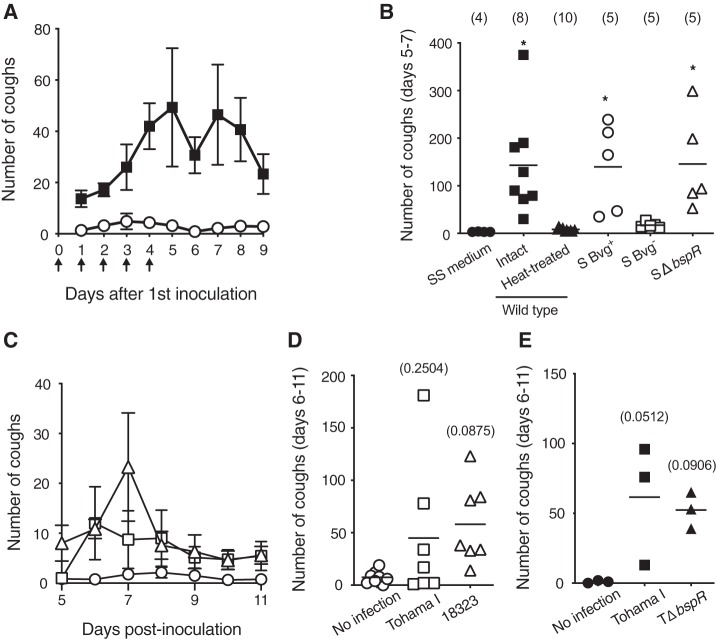
Cough production of rats intranasally inoculated with cell lysates of B. bronchiseptica (A and B) and B. pertussis (C to E). (A) Rats were intranasally inoculated with 100 µg of bacterial cell lysates (filled squares) or SS medium (open circles) in 100-µl portions every day from days 0 to 4 (arrows). The numbers of coughs were counted for 5 min/rat/day on indicated days. Each plot represents the means ± standard error (*n* = 6). (B) In independent experiments, rats were similarly inoculated with each bacterial cell lysate from wild-type S798, S Bvg^+^, S Bvg^–^, or SΔ*bspR* strains. An aliquot of wild-type lysate was incubated at 56°C for 1 h (heat treated). Coughs by rats were recorded for 10 min/rat/day for 3 days from days 5 to 7. Each horizontal bar represents the mean. The number of rats in each group is shown in parentheses. (C to E) Rats were intranasally inoculated with 10^8^ CFU of B. pertussis strains Tohama I (open squares, *n* = 7) and 18323 (open triangles, *n* = 7) or in SS medium without the bacteria (open circles, *n* = 8). (C) The numbers of coughs were counted for 5 min/rat/day on the indicated days. Each plot represents the means ± the standard errors. (D) The total numbers of coughs by each rat for 6 days from days 6 to 11 are shown. (E) Cough production by a *bspR*-deficient B. pertussis mutant. Rats (*n* = 3) were intranasally inoculated with 10^8^ CFU of B. pertussis Tohama I wild-type or a *bspR*-deficient mutant (TΔ*bspR*) strains. Coughs of rats were counted for 5 min/rat/day for 6 days from days 6 to 11 postinoculation, and the total numbers of coughs are shown. (D and E) Each horizontal bar represents the mean. Symbols for significant levels and actual *P* values in parentheses indicate comparison with the control “SS medium” (B) or “No infection” (D and E).

Rat coughing was also observed by intranasal administration of bacterial lysates (Fig. 4A and B). The lysates from the wild-type strain and a Bvg^+^-locked (S Bvg^+^) mutant in which the BvgAS system is constitutively active caused coughing, but a Bvg^–^-locked (S Bvg^–^) mutant in which the BvgAS system is dysfunctional did not. Heat treatment at 56°C for 1 h abolished the cough-producing ability of the lysates. These results revealed that the direct factor causing cough (the cough factor) is heat labile and produced in the Bvg^+^ phase. Unexpectedly, the lysates from SΔ*bspR* caused coughing to a similar extent as those from the wild-type strain, suggesting that *bspR* (*btrA*) does not encode the cough factor, and the production of the cough factor was likely regulated by BspR/BtrA in the host body but not in *in vitro* culture. Previous reports suggested that BspR/BtrA takes part in complex gene expression systems, and its regulatory function is modulated by intermediate accessary factors (22, 25). Therefore, it is not surprising that the phenotype of BspR/BtrA-deficient mutants differs between *in vitro* and *in vivo*.

Our rat model also exhibited coughing in response to B. pertussis infection, as well as in response to B. bronchiseptica infection (Fig. 4C to E). B. pertussis produces BspR/BtrA, which shares 98.4% identity in amino acid sequence with B. bronchiseptica (22). However, a *bspR* (*btrA*) (BP2233)-deficient mutant (TΔ*bspR*) of B. pertussis Tohama I still induced rat coughing (Fig. 4E), implying that the regulatory network that involves BspR/BtrA and the cough factor production in B. pertussis may be different from those in B. bronchiseptica, as previously reported (22). It is also possible that the cough factor of B. pertussis may be different from that of B. bronchiseptica, as discussed later. We consider that the rat model is not sufficient for deep analyses of cough production caused by B. pertussis for the following reasons: a large number of B. pertussis organisms was required, the frequencies of coughs were lower than those caused by B. bronchiseptica, and B. pertussis was eradicated from tracheas by 9 days after inoculation (data not shown). In addition, some rats did not exhibit coughing after B. pertussis inoculation, which often resulted in a lack of significance on statistical analyses (Fig. 4D and E).

## DISCUSSION

We used B. bronchiseptica, which naturally infects a wide variety of mammals, instead of B. pertussis, and succeeded in developing a simple and useful cough model in rats. Rats exhibited coughing after intranasal inoculation of different strains of B. bronchiseptica (RB50 and S798) and B. pertussis (Tohama I and 18323), suggesting that rat coughing is a common response to *Bordetella* infection; however, in the case of B. pertussis infection, the levels of cough varied among rats, as described in Results. Other groups previously reported rats that were experimentally infected with B. bronchiseptica, but they did not focus on coughing (20, 21, 26). In addition to the combination of rats with B. bronchiseptica, modern devices, such as a video camera and a personal computer to record coughing, allowed us to count the number of coughs as objectively as possible.

By comparing the cough numbers, we examined bacterial factors responsible for cough production (the cough factor). The present results demonstrated that known major virulence factors, such as adenylate cyclase toxin, dermonecrotic toxin, and type III-secreted effectors, are not involved in cough induced by B. bronchiseptica infection. In addition, we found that BspR/BtrA regulate the production of the cough factor. BspR/BtrA was recently reported as an anti-σ factor, which antagonizes BtrS, an extracytoplasmic function sigma factor, and is involved in the expression of a wide variety of genes in B. bronchiseptica and B. pertussis (22, 27). The expression of BspR/BtrA was regulated by the BvgAS regulatory system, upregulated in the Bvg^+^ phase, and secreted via the type III secretion system (22, 23, 28). We isolated Cgh^–^, which has a reduced ability to produce cough because of a frameshift mutation at Leu^85^ of BspR/BtrA consisting of 191 amino acid residues. BspR/BtrA is reported to comprise the N-terminal domain carrying the type III secretion signal, the central domain binding to BtrS, and the C-terminal domain required for transcriptional regulation (22, 28). Therefore, BspR/BtrA of Cgh^–^ is considered to lack the central and C-terminal domains, which are essential to antagonize the function of BtrS, and is thus unable to properly regulate the expression of downstream genes.

The *Bordetella* cough factor has not been characterized to date, but the present and previous studies provide some clues, albeit controversial. On one hand, the previous rat coughing model revealed that *B. parapertussis* and a pertussis toxin-deficient mutant of B. pertussis were inactive in cough production (9–13). Immunization with pertussis toxoid at least partially prevented coughing induced by the bead-embedded B. pertussis. In addition, it was reported that baboons immunized with acellular pertussis vaccines were protected from pertussis-associated symptoms, including cough production, but not from colonization (29). These results imply that pertussis toxin, which is not produced by *B. parapertussis*, is essential for cough production. On the other hand, there is the argument that the cough factor should be shared by the classical *Bordetella* because B. parapertussis and B. bronchiseptica do not produce pertussis toxin but cause cough in infected animals, including humans, rabbits, dogs, and pigs (16, 18, 30, 31). In particular, *B. parapertussis*-induced coughing is often associated with paroxysms indistinguishable from B. pertussis*-*induced coughing. The present study also demonstrates that pertussis toxin, even if produced by B. bronchiseptica, is not involved in cough production. These observations are inconsistent with the idea that pertussis toxin is the cough factor. Alternatively, it is possible that the cough factor is different in each *Bordetella* species.

In addition to the pertussis toxin, the present study provided insight into the nature of the cough factor. The cough factor is heat labile and expressed in the Bvg^+^ phase but not in the Bvg^–^ phase, similar to other major virulence factors of *Bordetella*. However, none of the major virulence factors, such as adenylate cyclase toxin, dermonecrotic toxin, and type III effectors, are the cough factor. BspR/BtrA of B. bronchiseptica may regulate the production of the cough factor in the course of infection, but not in *in vitro* culture, whereas that of B. pertussis is unlikely involved in cough production. Our rat model, which is easy to use, may help further analyses on the mechanism of the *Bordetella*-induced coughing.

## MATERIALS AND METHODS

### Bacterial strains.

Strains and derivatives of *Bordetella* used in this study are listed in Table 1 . B. bronchiseptica strain RB50 (32) was provided by P. A. Cotter, University of California, and B. bronchiseptica strain S798 (*ΔbscN* and *ΔbspR*) were provided by A. Abe, Kitasato University. B. bronchiseptica strain S798 and B. pertussis strains Tohama I and 18323 were maintained in the laboratory. B. pertussis and B. bronchiseptica were grown in Stainer-Scholte (SS) medium (33) or on Bordet-Gengou agar (Becton Dickinson, Franklin Lakes, NJ) containing 0.4% (wt/vol) polypeptone or hipolypeptone (Wako Pure Chemical Industries, Ltd., Japan), 0.8% glycerol, 20% defibrinated horse blood, and 10 µg/ml ceftibuten (BG plate). The number of bacterial cells was calculated from the correlation coefficient, 1 optical density at 650 nm (OD_650_) = 3.3 × 10^9^ cells/ml, which was obtained from a calibration curve with known concentrations of the bacterial cells versus OD_650_ values. Escherichia coli used for all genetic experiments was grown on Luria-Bertani (LB) agar or broth. E. coli strains DH5α λpir and HB101 harboring pRK2013 (34) were gifts from K. Minamisawa, Tohoku University. The growth media were supplemented with antibiotics when necessary at the following concentrations: gentamicin, 10 µg/ml; ceftibuten, 10 µg/ml; ampicillin, 50 µg/ml; kanamycin, 10 or 50 µg/ml; streptomycin, 10 µg/ml; and chloramphenicol, 10 µg/ml.

**TABLE 1:** Bacterial strains used in this study

Strain	Description	Source or reference
B. bronchiseptica		
RB50	Wild type	32
S798	Wild type	39
RΔ*dnt*	RB50 derivative, *dnt*::Cm^r^	This study
RΔ*cyaA*	RB50 derivative, *cyaA*::Cm^r^	This study
Cgh^–^	Spontaneous mutant of RB50, having a single base deletion in *bspR*	This study
RΔ*07570*	RB50 derivative, *BB_RS07570*::Cm^r^	This study
RΔ*14645*	RB50 derivative, *BB_RS14645*::Cm^r^	This study
Cgh^–^:*bspR*	Cgh^–^ derivative complemented with *bspR*	This study
R*bspR*^FS^	RB50 derivative, having a single base deletion in *bspR* equivalent to Cgh^–^	This study
RΔ*bspR*	RB50 derivative, *bspR*::Cm^r^	This study
RΔ*bspR*_85-C_	RB50 derivative with the C-terminally truncated BspR	This study
RΔ*bspR*: pMock	RΔ*bspR* derivative carrying pMIN136TDE-P*cyaA*	This study
RΔ*bspR*: p*bspR*	RΔ*bspR* derivative carrying pMIN136TDE-P*cyaA-*P*_bspR_-bspR*	This study
RΔ*bspR*:p*bspR*_ATG1_	RΔ*bspR* derivative carrying pMIN136TDE-P*cyaA-*P*_bspR_-bspR*_ATG1_	This study
RΔ*bspR*: p*bspR*_ATG2_	RΔ*bspR* derivative carrying pMIN136TDE-P*cyaA-*P*_bspR_-bspR*_ATG2_	This study
RΔ*ptxptl*	RB50 derivative, *ptxA-ptlH*::Cm^r^	This study
Δ*bscN*	S798 derivative, Δ*bscN*	23
Δ*bspR*	S798 derivative, Δ*bspR* (in-frame deletion)	23
SΔ*bspR*	S798 derivative, *bspR*::Cm^r^	This study
S Bvg^+^	S798 derivative producing BvgS in which Arg was replaced with His at amino acid position 570 (Bvg^+^ phase-locked mutant)	This study
S Bvg^–^	S798 derivative, of which BvgS was deleted from amino acid positions 541 to 1020 (Bvg^–^ phase-locked mutant)	This study
		
B. pertussis		
Tohama I	Wild type	24
TΔ*bspR*	Tohama I derivative, *bspR*::Cm^r^	This study
18323	Wild type	17

### Construction of mutant strains.

Mutant strains were generated as follows using the plasmid vectors and primers listed in Tables S1 and S2 in the supplemental material, respectively. Genes constructed and introduced by conjugation as described below were integrated into the corresponding regions of parental strains by two-step homologous recombination. The integration of introduced genes was confirmed by sequencing. B. bronchiseptica RΔ*dnt*, RΔ*cyaA*, RΔ*bspR*, *R*Δ*07570*, *R*Δ*14645*, *R*Δ*ptxptl*, SΔ*bspR*, S Bvg^+^, and S Bvg^–^ strains and B. pertussis TΔ*bspR* were generated by a previously described method (35). In the construction of RΔ*dnt*, pDONR201 (Invitrogen) and pABB-CRS2 (36), gifts from A. Abe, Kitasato University, were used as the cloning and positive suicide vectors, respectively. A 1.3-kbp DNA fragment containing a 5′ region of the *dnt* gene was amplified by PCR with the primers DNT-F1 and DNT-R1 using B. pertussis Tohama I genomic DNA as a template because there are only two nucleotide differences in the region between B. bronchiseptica RB50 and B. pertussis Tohama I. The resulting PCR product was cloned into pDONR201 to obtain *dnt*-pDONR using the BP reaction in the Gateway cloning system (Invitrogen). Then, ∼1-kbp DNA fragments (named DNT-U and DNT-D) were amplified by PCR using circular *dnt*-pDONR as the template, and the combination of primers DNT-U-F and DNT-U-R, as well as another combination of primers, DNT-D-F and DNT-D-R. DNT-U and DNT-D were digested with ApaI/KpnI and KpnI/BglII, respectively, and cloned into ApaI- and BglII-digested pDONR201 to obtain RB50 Δ*dnt*-pDONR, which contained a 250-bp deletion in the 5′ region of the *dnt* gene. RB50 Δ*dnt*-pDONR was mixed with pABB-CRS2 to obtain RB50 Δ*dnt*-pABB-CRS2 using the Gateway cloning system. A fragment of the chloramphenicol-resistant gene (Cm^r^) with the KpnI sites at both ends, which originated from pKD3 (37), was inserted into the KpnI site of RB50 Δ*dnt*-pABB-CRS2. The resultant plasmid (RB50 Δ*dnt*-pABB-CRS2::Cm^r^) was introduced into E. coli SM10 λpir and transconjugated into B. bronchiseptica RB50 by biparental conjugation. The resulting mutant strain was designated RΔ*dnt*.

10.1128/mSphere.00093-19.1 TABLE S1 Plasmids used in this study. Download Table S1, DOCX file, 0.02 MB. Copyright © 2019 Nakamura et al. 2019 Nakamura et al. This content is distributed under the terms of the Creative Commons Attribution 4.0 International license.

10.1128/mSphere.00093-19.2 TABLE S2 Primers used in this study. Download Table S2, DOCX file, 0.02 MB. Copyright © 2019 Nakamura et al. 2019 Nakamura et al. This content is distributed under the terms of the Creative Commons Attribution 4.0 International license.

B. bronchiseptica Cgh^–^:*bspR*, R*bspR*^FS^, and RΔ*bspR*_85-C_ were constructed as described previously with slight modifications (38). An ∼1-kbp fragment of DNA including *bspR* or *bspR*^FS^ with a single base deletion at base position 253 was amplified with genomic DNA from RB50 wild-type or Cgh^–^ strains, respectively, as the template by PCR with primers BspR-F1 and BspR-R1. Each DNA fragment was inserted into pABB-CRS2-Gm, which was used for the preparation of RB50 *bspR*-pABB-CRS2-Gm and RB50 *bspR*^FS^-pABB-CRS2-Gm. BB_RS19685-pABB-CRS2-Gm, used for the generation of *R*Δ*19685:19685*, was constructed by inserting an ∼2.4-kbp fragment of DNA, including *BB_RS19685* into pABB-CRS2-Gm. For construction of RB50 *bspR1-84*-pABB-CRS2-Gm, a 2.4-kbp DNA fragment containing the *bspR* gene was amplified by PCR with the primers RB50-BspR-U-F and RB50-BspR-D-R using B. bronchiseptica RB50 genomic DNA as a template. The PCR product was inserted using an In-Fusion HD cloning kit (Clontech Laboratories, Inc.) into the SmaI site of pCR4blunt-CRS2, which originated from pCR4blunt-TOPO and contains sequences overlapping those of pABB-CRS2-Gm. The resultant plasmid was designated RB50 *bspR*-pCR4blunt-CRS2. Subsequently, inverse PCR was carried out with RB50 *bspR*-pCR4blunt-CRS2 as the template, and the primers BspR-F2 and BspR-R2 containing a stop codon at the 5′ end. The final PCR products were phosphorylated with T4 Polynucleotide Kinase (TaKaRa Bio, Inc., Tokyo, Japan) and self-ligated to obtain RB50 *bspR1-84*-pCR4blunt-CRS2 encoding the N-terminal 84 amino acid residues of BspR. A 2.1-kbp DNA fragment containing the truncated *bspR* gene was amplified by PCR using RB50 *bspR1-84*-pCR4blunt-CRS2 as the template and the primers RB50-BspR-U-F and RB50-BspR-D-R and then inserted into the SmaI site of pABB-CRS2-Gm using the In-Fusion HD cloning kit. The resultant plasmid, *bspR1-84*-pABB-CRS2-Gm, was introduced into E. coli strain DH5α λpir and, subsequently, B. bronchiseptica strain RB50 by triparental conjugation with a helper E. coli HB101/pRK2013 strain.

Plasmids for complementation experiments were generated as below. The promoter region of *cyaA* and the terminator region of *rrnB* were amplified by PCR with a combination of primers, P*_cyaA_*-F and P*_cyaA_*-R, and RB50 genomic DNA as the template, and another combination of primers, rrnBT1T2-F and rrnBT1T2-R, plus pKK232-8 (Addgene) as the template, respectively. The amplified fragments were introduced into pMIN136TDE digested with EcoRV and SpeI using the In-Fusion HD cloning kit. The resultant plasmid was designated pMIN136TDE-P*cyaA*. The fragment including *bspR* and its promoter was amplified by PCR with PbspR-bspR-F and PbspR-bspR-R primers, using RB50 genomic DNA as the template, and introduced into pMIN136TDE-P*cyaA* digested with NdeI and NcoI. The obtained plasmid was designated pMIN136TDE-P*cyaA*-P*_bspR_*-*bspR* and used as a template for inverse PCR with the primers bspR_ATG2_-F and bspR_ATG2_-R and the primers bspR_ATG1_-F and bspR_ATG1_-R. Finally, pMIN136TDE-P*cyaA*-P_bspR_-bspR_ATG2_ and pMIN136TDE-P*cyaA*-P_bspR_-bspR_ATG1_ were obtained.

### Animal experiments.

The bacteria recovered from colonies on BG plates were suspended in SS medium to an OD_650_ value of 0.1 or 0.2, followed by incubation at 37°C for a sufficient period with shaking to give the desired bacterial concentration. Female Wistar rats (3 or 4 weeks old; Japan SLC, Inc., Japan) were anesthetized with ether, a small amount of pentobarbital, or a mixture of medetomidine (Kyoritsu, Japan), midazolam (Taiyo, Japan), and butorphanol (Meiji Seika Pharma, Japan) at final doses of 0.3, 2, and 5.0 mg/kg (body weight), respectively, and intranasally inoculated with B. bronchiseptica (1 × 10^3^, 1 × 10^2^, or 10 CFU) or B. pertussis (1 × 10^8^ CFU) in 10 or 50 µl of SS medium using a micropipette with a needle-like tip. The bacterial culture prepared for inoculation into rats was spread onto BG plates, and the bacterial number in the inoculum was confirmed by counting the CFU. On the days postinoculation, rats were euthanized with pentobarbital and the tracheas were removed, weighed, minced in 200 µl of Dulbecco phosphate-buffered saline (PBS), and strained with a Biomasher (Funakoshi Co., Ltd., Japan). The resultant tissue extracts were serially diluted in Dulbecco PBS and plated on BG plates. The bacteria on the plate were cultivated at 37°C for 2 days, and the number of CFU was determined.

For preparation of bacterial lysates, B. bronchiseptica S798 wild-type or mutant strains at an OD_650_ of 0.1 in SS medium were cultivated for 5 h, and the cultured cells were collected by centrifugation at 2,300 × *g* for 15 min. The bacteria resuspended in SS medium were disrupted by five 2-min periods of sonication with Bioruptor (Cosmo Bio Co., Ltd., Japan). The sonicated suspension was centrifuged at 14,000 × *g* for 5 min, and the supernatant was filtered through a 0.22-μm-pore size filter (Millex GV; Merck Millipore Co., Germany). The resultant bacterial lysates were intranasally inoculated at 100 µg/100 µl into 3-week-old female Wistar rats anesthetized with isoflurane.

The numbers of rat coughs were calculated as follows. Rats inoculated with bacteria or bacterial lysates were isolated individually in a disposable clear plastic cage, which was covered with a plastic cardboard lid, and recorded with a video camera (Canon HD ivisHF21; Canon, Inc., Japan) equipped with a stereo microphone (AT9900; Audio-Technica Co., Japan) every day for 5 min a day or every other day for 10 min a day during the indicated period. The recorded data were processed with Adobe Premier Pro CS5.5 (Adobe Systems, Inc.) on a computer and displayed as movies, along with sound waveforms. Coughs were analyzed by characteristic waveforms and coughing postures of rats and counted by an observer who was blinded to the experiments.

All animal experiments were approved by the Animal Care and Use Committee of the Research Institute for Microbial Disease, Osaka University, and carried out according to the Regulations on Animal Experiments at Osaka University.

### Others.

For SDS-PAGE, cultures of *Bordetella* were obtained after incubation in SS medium until OD_650_ values reached 1.5 to 2.0. The culture supernatants and bacterial cells were separated by centrifugation, and the former were mixed with trichloroacetic acid (TCA) at a final concentration of 5% and allowed to stand on ice for 15 min. The resultant precipitates were sequentially washed with 5% TCA and cold acetone, suspended in 2-fold-concentrated SDS-PAGE sample buffer, and subjected to SDS-PAGE (12.5% gel), followed by staining with Coomassie brilliant blue R-250. The bacterial cells were lysed with sonic treatment, suspended in SDS-PAGE sample buffer, and subjected to SDS-PAGE (15% gel). After electrophoresis, proteins in gels were transferred onto a polyvinylidene difluoride membrane (Millipore), and the membrane was blocked with 5% skim milk. The membrane was then incubated with polyclonal anti-BspR antibody, which was provided by A. Abe, Kitasato University, followed by peroxidase-conjugated goat anti-rabbit IgG (Jackson Immune Research Laboratories, Inc., West Grove, PA). The target proteins were visualized on Fuji Medical film (Fujifilm, Japan) with an enhanced chemiluminescence system in accordance with the manufacturer’s instructions (GE Healthcare).

The complete genome sequence of the B. bronchiseptica derivative Cgh^–^ was determined as previously described (39) and then compared to that of strain RB50 (NC_002977). In this analysis, we found 37 single nucleotide polymorphisms (SNPs). This number of SNPs was somewhat large, suggesting that some of these SNPs had already occurred in the parental strain of Cgh^–^, the RB50 clone used in our laboratory. Therefore, we sequenced SNP-containing regions of Cgh^–^ and the parental strain by Sanger sequencing and compared the sequences between the two strains to confirm all SNPs. This analysis revealed that 34 of the 37 SNPs that we initially identified were already present in our RB50 clone or were sequencing errors in the genome sequences of RB50 or Cgh^–^ and that the remaining three SNPs were truly specific to Cgh^–^.

### Statistical analysis.

Statistical analyses were performed using Prism 7 (GraphPad Software). One-way analysis of variance and Tukey’s multiple-comparison test were used for cough numbers. For the CFU numbers, the Mann-Whitney test and Kruskal-Wallis test with Dunn’s multiple-comparison test were carried out for comparison between two test groups and among more than two test groups, respectively. Significance is expressed as *P* values (*, *P* < 0.05; **, *P* < 0.01; ***, *P* < 0.001; ****, *P* < 0.0001).
