# Supplementary material for: BspR/BtrA, an Anti-σ Factor, Regulates the Ability of Bordetella bronchiseptica To Cause Cough in Rats
Source: mSphere. 2019 Apr 24;4(2):e00093-19. doi: 10.1128/mSphere.00093-19 (PMC6483047; doi:10.1128/mSphere.00093-19)
Supplement: FIG S1 [file mSphere.00093-19-sf001.pdf]

A

```
 -100       -90       -80       -70       -60       -50       -40       -30
atggaaccgaagagccgaaagcgtcacacagtgcatattccatattccatgttcccgctccgtagaacgcgatacaccca

  -20       -10         1        10        20        30        40        50
aggattcgagacaacggcccaccgcatgaacttccagatcccacccgctttacctgctttggagcttgatgtctttgcgc
                         M  N  F  Q  I  P  P  A  L  P  A  L  E  L  D  V  F  A  R
                         1                       10

   60        70        80        90       100       110       120       130
gcgccgccagccaaggagagaccctttatgtcaccaaagcaggcgagcagttccaggtcatcgcatccggcacgacgcca
 A  A  S  Q  G  E  T  L  Y  V  T  K  A  G  E  Q  F  Q  V  I  A  S  G  T  T  P
 20                            30                            40

  140       150       160       170       180       190       200       210
tcagggcgcaacgtatcctgggtcgccaccgacgaggacacgcttgtcatgttttccagcgcgctggcgctggcctacgg
S  G  R  N  V  S  W  V  A  T  D  E  D  T  L  V  M  F  S  S  A  L  A  L  A  Y  G
            50                            60                            70

  220       230       240       250 ↓     260       270       280       290
cacgggaatcgcccgcgccgtcgccaaggagctcgatctgcacgcggccccgacgacatcgctgtcggcgcgtgtcgtca
 T  G  I  A  R  A  V  A  K  E  L  D  L  H  A  A  P  T  T  S  L  S  A  R  V  V  T
                       80                      90
                                       C  T  R  P  R  R  H  R  C  R  R  V  S  S

  300       310       320       330       340       350       360       370
cgcgagccgtcgacatggcggaaacctcacgccacgccctgcagggcgtggatttccttaccttcctgtcctggtcggcg
 R  A  V  D  M  A  E  T  S  R  H  A  L  Q  G  V  D  F  L  T  F  L  S  W  S  A
 100                           110                           120
R  E  P  S  T  W  R  K  P  H  A  T  P  C  R  A  W  I  S  L  P  S  C  P  G  R  R

  380       390       400       410       420       430       440       450
cgcgccgacaccgccggcttccgacaagtctgtcacgacaccggtgtctctcccgatcagatatccggaacgttgcgtgc
R  A  D  T  A  G  F  R  Q  V  C  H  D  T  G  V  S  P  D  Q  I  S  G  T  L  R  A
            130                           140                           150
 A  P  T  P  P  A  S  D  K  S  V  T  T  P  V  S  L  P  I  R  Y  P  E  R  C  V  P

  460       470       480       490       500       510       520       530
cacgatcgacgaaagcatgcagcagcgcttcgcatccgccgcacaatcaggtaaggcgccggtatccgcccatacggcgc
 T  I  D  E  S  M  Q  Q  R  F  A  S  A  A  Q  S  G  K  A  P  V  S  A  H  T  A  Q
                       160                           170
 R  S  T  K  A  C  S  S  A  S  H  P  P  H  N  Q  V  R  R  R  Y  P  P  I  R  R

  540       550       560       570
aagaatggctgcgcgaggtccttgcgcaccacctgatgtag
 E  W  L  R  E  V  L  A  H  H  L  M  *
 180                           190
K  N  G  C  A  R  S  L  R  T  T  *
```

B

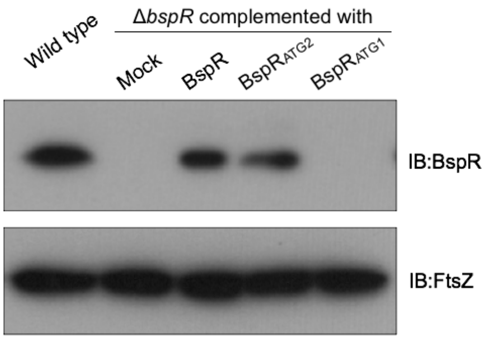

Figure S1: Determination of the start codon of *bspR/btrA*. (A) Base and deduced amino-acid sequences of *bspR/btrA*. Two ATGs are present 105 bp apart from each other in the 5' region of *bspR/btrA* ORF (underlines). The stop codons are indicated by asterisks. Cgh⁻ has a cytosine deletion at the position indicated by the arrow, resulting in translational frameshift at Leu^{85} as shown in blue letters. (B) *B. bronchiseptica* strain RB50 Δ*bspR* was transformed with pMIN136TDE-P*cyaA*-P_{bspR}-*bspR*, pMIN136TDE-P*cyaA*-P_{bspR}-$bspR_{ATG1}$ ($BspR_{ATG1}$), or pMIN136TDE-P*cyaA*-P_{bspR}-$bspR_{ATG2}$ ($BspR_{ATG2}$), and cultivated for 5 h in SS medium. The whole cell lysates were obtained and subjected to SDS-PAGE followed by immunoblotting for BspR/BtrA as described in Materials and Methods. The above-mentioned plasmids express BspR under control of the promoters of *cyaA* and *bspR/btrA*. The former plasmid encodes intact *bspR*. The latter two plasmids encode *bspR/btrA* with ACG in place of the second ATG and the first ATG, respectively. pMIN136TDE-P*cyaA* was used as a vector control. Note that $BspR_{ATG2}$ but not $BspR_{ATG1}$ produced BspR as detected by immunoblotting, indicating that the second ATG functions as the start codon for BspR/BtrA. FtsZ was detected by anti-FtsZ as an internal control.
