# Supplementary material for: BspR/BtrA, an Anti-σ Factor, Regulates the Ability of Bordetella bronchiseptica To Cause Cough in Rats
Source: mSphere. 2019 Apr 24;4(2):e00093-19. doi: 10.1128/mSphere.00093-19 (PMC6483047; doi:10.1128/mSphere.00093-19)
Supplement: TABLE S1 [file mSphere.00093-19-st001.docx]

| Table S1. Plasmids used in this study | | |
| --- | --- | --- |
| **Plasmids** | **Description** | **Source or reference** |
| pKK232-8 | Cm^r^, cloning vector | Addgene |
| pKD3 | Cm^r^, cloning vector | (1) |
| pRK2013 | Km^r^, RK-2 derivative with ColE1 replicon containing *tra*, helper plasmid for conjugative transfer | (2) |
| pDONR201 | Km^r^, cloning vector | Invitrogen |
| pCR4blunt-CRS2 | Amp^r^, pCR4blunt-TOPO-derived cloning vector | Laboratory collection |
| pABB-CRS2 | Amp^r^, R6K-derived suicide vector | (3) |
| pABB-CRS2-Gm | Gm^r^, R6K-derived suicide vector | (4) |
| *dnt*-pDONR | Tohama I *dnt* (1-1537) cloned into pDONR | This study |
| ∆*dnt*-pDONR | Tohama I *dnt* deletion cloned into pDONR | This study |
| ∆*dnt*-pABB-CRS2 | Tohama I *dnt* deletion cloned into pABB-CRS2 | This study |
| ∆*dnt*-pABB-CRS2::Cm^r^ | Cm^r^, Tohama I *dnt* deletion cloned into pABB-CRS2 | This study |
| ∆*cyaA*-pABB-CRS2-Gm | RB50 *cyaA* deletion cloned into pABB-CRS2-Gm | This study |
| ∆BB_RS07570-pABB-CRS2-Gm | RB50 *BB_RS07570-*deletion cloned into pABB-CRS2-Gm | This study |
| ∆BB_RS14645-pABB-CRS2-Gm | RB50 *BB_RS14645-*deletion cloned into pABB-CRS2-Gm | This study |
| *bspR*-pABB-CRS2-Gm | RB50 *bspR* cloned into pABB-CRS2-Gm | This study |
| *bspR^FS^*-pABB-CRS2-Gm | RB50 *bspR* single base deletion cloned into pABB-CRS2-Gm | This study |
| ∆*bspR*-pABB-CRS2-Gm | RB50 *bspR* deletion cloned into pABB-CRS2-Gm | This study |
| *bspR*-pCR4blunt-CRS2 | RB50 *bspR* cloned into pCR4blunt-CRS2 | This study |
| *bspR*_1-84_-pCR4blunt-CRS2 | RB50 truncated *bspR* (1-252) cloned into pCR4blunt-CRS2 | This study |
| *bspR*_1-84_-pABB-CRS2-Gm | RB50 truncated *bspR* (1-252) cloned into pABB-CRS2-Gm | This study |
| ∆*ptxptl*-pABB-CRS2-Gm | RB50 *ptx* and *ptl* operon deletion cloned into pABB-CRS2-Gm | This study |
| pMIN136TDE | Km^r^, cloning vector containing oriT(RK2) and *parDE* | (5) |
| pMIN136TDE-P*_cyaA_* | pMIN136TDE containing the *cyaA* promoter and rrnBT1BT2 terminator | This study |
| pMIN136TDE-P*_cyaA_-*P*_bspR_-bspR* | pMIN136TDE-P*cyaA* carrying *bspR* with the *bspR* promoter | This study |
| pMIN136TDE-P*_cyaA_-*P*_bspR_-bspR*_ATG1_ | pMIN136TDE-P*cyaA* carrying *bspR*_ATG2_ with the *bspR* promoter | This study |
| pMIN136TDE-P*_cyaA_-*P*_bspR_-bspR*_ATG2_ | pMIN136TDE-P*cyaA* carrying *bspR*_ATG1_ with the *bspR* promoter | This study |
| ∆S798_*bspR*-pABB-CRS2-Gm | S798 *bspR* deletion cloned into pABB-CRS2-Gm | This study |
| *bvgS*-C3—pABB-CRS2-Gm | S798 *bvgS*-C3 mutation cloned into pABB-CRS2-Gm | This study |
| ∆*bvgS*-pABB-CRS2-Gm | S798 *bvgS* deletion cloned into pABB-CRS2-Gm | This study |
| ∆BP2233-pABB-CRS2-Gm | Tohama I *BP2233* deletion cloned into pABB-CRS2-Gm | This study |

1. Datsenko KA, Wanner BL. 2000. One-step inactivation of chromosomal genes in *Escherichia coli* K-12 using PCR products. Proc Natl Acad Sci USA 97:6640–6645.

2. Figurski DH, Helinski DR. 1979. Replication of an origin-containing derivative of plasmid RK2 dependent on a plasmid function provided in trans. Proc Natl Acad Sci USA 76:1648–1652.

3. Sekiya K, Ohishi M, Ogino T, Tamano K, Sasakawa C, Abe A. 2001. Supermolecular structure of the enteropathogenic *Escherichia coli* type III secretion system and its direct interaction with the EspA-sheath-like structure. Proc Natl Acad Sci USA 98:11638–11643.

4. Okada K, Ogura Y, Hayashi T, Abe A, Kuwae A, Horiguchi Y, Abe H. 2014. Complete Genome Sequence of *Bordetella bronchiseptica* S798, an Isolate from a Pig with Atrophic Rhinitis. Genome Announcements 2:e00436–14–e00436–14.

5. Nishikawa S, Shinzawa N, Nakamura K, Ishigaki K, Abe H, Horiguchi Y. 2016. The bvg-repressed gene brtA, encoding biofilm-associated surface adhesin, is expressed during host infection by *Bordetella bronchiseptica*. Microbiology and Immunology 60:93–105.
