# Supplementary material for: BspR/BtrA, an Anti-σ Factor, Regulates the Ability of Bordetella bronchiseptica To Cause Cough in Rats
Source: mSphere. 2019 Apr 24;4(2):e00093-19. doi: 10.1128/mSphere.00093-19 (PMC6483047; doi:10.1128/mSphere.00093-19)
Supplement: TABLE S2 [file mSphere.00093-19-st002.docx]

| Table S2. Primers used in this study | | | |
| --- | --- | --- | --- |
| **Name** | **Sequence (5’-3’) *** | **Template** | **For construction of** |
| DNT-F1  DNT-R1 | GGGGACAAGTTTGTACAAAAAAAAGCAGGCTCACCG-  GCAAACTGTTCCTGT  GGGGACCACTTTGTACAAGAAAGCTGGGTCAACGGT-  GCGCATATACCCT | Tohama I genomic DNA | *dnt*-pDONR |
| DNT-U-F  DNT-U-R | CGTTATCCCCTGATTCTGTG  CGGGGTACCCCGTCAGTAGCCTATAAGCGCCATGTC | *dn*t-pDONR | *∆dnt*-pDONR |
| DNT-D-F  DNT-D-R | CGGGGTACCCCGGTGGTTGATCGCACTCGATGT  CCAGTTTAGTCTGACCATCT | *dnt*-pDONR | *∆dnt*-pDONR |
| RB50-ACT-U-F  RB50-ACT-U-R | GATCCGAGCTCTCCCTGGCCGAGTACATGCAGA  TTTAGCTTCCTTAGCTCTGATGCGATTGCTGCATGT | RB50 genomic DNA | ∆*cyaA*-pABB-CRS2-Gm |
| RB50-ACT-D-F  RB50-ACT-D-R | CGCGCCATTTAAATGGCTGTCAACTGGCGCTGA  ATTTGTGGAATTCCCGAAAATAGCAAGGCAGCG | RB50 genomic DNA | ∆*cyaA*-pABB-CRS2-Gm |
| BB_RS07570-U-F  BB_RS07570-U-R | GATCCGAGCTCTCCCATGACTGGCATACAAAAGAAGC  TTAGCTTCCTTAGCTGAAGGTCAGAAACTTCATTTCAG | RB50 genomic DNA | ∆BB_RS07570-pABB-CRS2-Gm |
| BB_RS07570-D-F  BB_RS07570-D-R | CGCGCCATTTAAATGAACGAAATCGTCGCTTGATTC  ATTTGTGGAATTCCCCTCTTCGACCGAGTGGAAC | RB50 genomic DNA | ∆BB_RS07570-pABB-CRS2-Gm |
| BB_RS14645-U-F  BB_RS14645-U-R | GATCCGAGCTCTCCCAATCGCTGCCGTACCAA  TTAGCTTCCTTAGCTGGCAATACGCTTTGCCAT | RB50 genomic DNA | ∆BB_RS14645-pABB-CRS2-Gm |
| BB_RS14645-D-F  BB_RS14645-D-R | CGCGCCATTTAAATGCAGAAAATGATCCATTGAATCCCA  ATTTGTGGAATTCCCATATCCACGTCGTTGCCG | RB50 genomic DNA | ∆BB_RS14645-pABB-CRS2-Gm |
| BspR-F1  BspR-R1 | GATCCGAGCTCTCCCACAACGCGCTGAATGCC  ATTTGTGGAATTCCCAGCGTATGGCCGACACT | RB50 genomic DNA | *bspR*-pABB-CRS2-Gm |
| BspR-F1  BspR-R1 | GATCCGAGCTCTCCCACAACGCGCTGAATGCC  ATTTGTGGAATTCCCAGCGTATGGCCGACACT | RB50 Cdm genomic DNA | *bspR^FS^*-pABB-CRS2-Gm |
| RB50-BspR-U-F  RB50-BspR-U-R | GATCCGAGCTCTCCCGACGAAATGTTCGCTGCGCGCGG  TTAGCTTCCTTAGCTTCGGCTCTTCGGTTCCATGCC | RB50 genomic DNA | ∆*bspR*-pABB-CRS2-Gm |
| RB50-BspR-D-F  RB50-BspR-D-R | CGCGCCATTTAAATGCGCACCACCTGATGTAGTCC  ATTTGTGGAATTCCCTTCAGCCCCGTTGCGGGCA | RB50 genomic DNA | ∆*bspR*-pABB-CRS2-Gm |
| RB50-BspR-U-F2  RB50-BspR-D-R | GATCCGAGCTCTCCCCCAGCGGGCTGTTGGCGAA  ATTTGTGGAATTCCCTTCAGCCCCGTTGCGGGCA | RB50 genomic DNA | *bspR*-pCR4blunt-CRS2 |
| BspR-F2  BspR-R2 | GCGCACCACCTGATGTAGTCC  TTAATCGAGCTCCTTGGCGACGG | *bspR*-pCR4blunt-CRS2 | *bspR*_1-84_-pCR4blunt-CRS2 |
| RB50-BspR-U-F2  RB50-BspR-D-R | GATCCGAGCTCTCCCCCAGCGGGCTGTTGGCGAA  ATTTGTGGAATTCCCTTCAGCCCCGTTGCGGGCA | *bspR*_1-84_-pCR4blunt-CRS2 | *bspR*_1-84_-pABB-CRS2-Gm |
| BB_RS19685-U-F  BB_RS19685-U-R | GATCCGAGCTCTCCCTGAACTTGCACCACGG  TTAGCTTCCTTAGCTGCGGGAAGGATTGAACATCG | RB50 genomic DNA | ∆BB_RS19685-pABB-CRS2-Gm |
| BB_RS19685-D-F  BB_RS19685-D-R | CGCGCCATTTAAATGCGCCGAGCGCTAGAAAA  ATTTGTGGAATTCCCTCGTCGGTGGCGACGTA | RB50 genomic DNA | ∆BB_RS19685-pABB-CRS2-Gm |
| BB_RS19685-U-F  BB_RS19685-D-R | GATCCGAGCTCTCCCTGAACTTGCACCACGG  ATTTGTGGAATTCCCTCGTCGGTGGCGACGTA | RB50 genomic DNA | BB_RS19685-pABB-CRS2-Gm |
| PcyaA-F  PcyaA-R | ATCGATAAGCTTGATATCATATTCCGTGTTGGGTGCGC  CGTTAACCATGGCATATGACATCCAGCACGTCGTATGA | RB50 genomic DNA | pMIN136TDE-P*cyaA* |
| rrnBT1T2-F  rrnBT1T2-R | ATGCCATGGTTAACGCGTATGGTAGTGTGGGGTCTCCC  ACCGTCTAGAACTAGTACGACAGGAAGAGTTTGTAGAAACG | pKK232-8 | pMIN136TDE-P*cyaA* |
| PbspR-bspR-F  PbspR-bspR-R | GACGTGCTGGATGTCATATGGCCGACACTTTGCGCA  ATACGCGTTAACCATGGGGAGAAACCATGCAAAGCG | RB50 genomic DNA | pMIN136TDE-P*cyaA-*P*_bspR_-bspR* |
| bspR_ATG2_-F  bspR_ATG2_-R | TTTGTCATGCCGGCACGGAACCGAAGAGCCG  CGGCTCTTCGGTTCCGTGCCGGCATGACAAA | pMIN136TDE-P*cyaA-*P*_bspR_*  *-bspR* | pMIN136TDE-P*cyaA-*P*_bspR_-bspR*_ATG1_ |
| bspR_ATG1_-F  bspR_ATG1_-R | CAACGGCCCACCGCACGAACTTCCAGATCCC  GGGATCTGGAAGTTCGTGCGGTGGGCCGTTG | pMIN136TDE-P*cyaA-*P*_bspR_*  *-bspR* | pMIN136TDE-P*cyaA-*P*_bspR_-bspR*_ATG2_ |
| S798-BspR-U-F  S798-BspR-U-R | GATCCGAGCTCTCCCGAACTGCGAATAGACGA  TTAGCTTCCTTAGCTCGGCTCTTCGGTTCCAT | S798 genomic DNA | ∆S798_*bspR*-pABB-CRS2-Gm |
| RB50-BspR-D-F  RB50-BspR-D-R | CGCGCCATTTAAATGCGCACCACCTGATGTAGTCC  ATTTGTGGAATTCCCTTCAGCCCCGTTGCGGGCA | S798 genomic DNA | ∆S798_*bspR*-pABB-CRS2-Gm |
| BB_RS19685-U-F  BB_RS19685-U-R | GATCCGAGCTCTCCCTGAACTTGCACCACGG  TTAGCTTCCTTAGCTGCGGGAAGGATTGAACATCG | S798 genomic DNA | ∆S798_3705-pABB-CRS2-Gm |
| BBS798_3705-D-F  BB_RS19685-D-R | CGCGCCATTTAAATGAGCGCTAGAAAAGGAAAAG  ATTTGTGGAATTCCCTCGTCGGTGGCGACGTA | S798 genomic DNA | ∆S798_3705-pABB-CRS2-Gm |
| S798-BvgS-C3-U  S798-BvgS-C3-D | GATCCGAGCTCTCCCAAGCTACTTCAACGACGTTC  ATTTGTGGAATTCCCTCAATTCGACGAGGCGG | S798 genomic DNA | bvg1-pABB-CRS2-Gm |
| S798-BvgS-C3-F  S798-BvgS-C3-R | GCCAGATCCGCCAGCACAAGCGGGCCGAGCG  CGCTCGGCCCGCTTGTGCTGGCGGATCTGGC | bvg1-pABB-CRS2-Gm | bvgS-C3-pABB-CRS2-Gm |
| S798-BvgS-U-F  S798-BvgS-D-R | GATCCGAGCTCTCCCACGCTGCATTACTTCCCATC  ATTTGTGGAATTCCCCTTACCGTCAGTACGTTCGATG | S798 genomic DNA | bvg2-pABB-CRS2-Gm |
| S798-BvgS-U-R  S798-BvgS-D-F | TTCGTTGCGGTAGGCGTA  ATCACCGATTGCAACATGCC | bvg2-pABB-CRS2-Gm | ∆bvgS-pABB-CRS2-Gm |
| BP2233-U-F  BP2233-U-R | GATCCGAGCTCTCCCGGGGACAATCGGCATCC  TTAGCTTCCTTAGCTAAACCCGACGGCATGCA | Tohama I genomic DNA | ∆BP2233-pABB-CRS2-Gm |
| BP2233-D-F  BP2233-D-R | CGCGCCATTTAAATGCGCACCACCTGGTGTAG  ATTTGTGGAATTCCCGTGGCTATCGAGGGCAA | Tohama I genomic DNA | ∆BP2233-pABB-CRS2-Gm |
| RB50-PtxPtl-U-F  RB50-PtxPtl-U-R | GATCCGAGCTCTCCCACCGGACCACAAGGCTCATG  TTAGCTTCCTTAGCTCTACTTCAGCTTGGCGCCGGTTT | RB50 genomic DNA | ∆*ptxptl*-pABB-CRS2-Gm |
| RB50-PtxPtl-D-F  RB50-PtxPtl-D-R | CGCGCCATTTAAATGCGCCTGCACGCATTGCGATT  ATTTGTGGAATTCCCTGGATCGCCATCAAGCTGCAG | RB50 genomic DNA | ∆*ptxptl*-pABB-CRS2-Gm |
| CmR-F  CmR-R | AGCTAAGGAAGCTAAAATGGAG  CATTTAAATGGCGCGCCTT | pKD3 | Chloramphenicol resistant gene |
|  | *Underlines indicate homologous regions for In-Fusion cloning. | |  |
